# Arabic as a home language in Sweden: family language practices and beliefs

**DOI:** 10.3389/fpsyg.2025.1719805

**Published:** 2025-12-16

**Authors:** Ute Bohnacker, Rima Haddad

**Affiliations:** Department of Linguistics & Philology, Uppsala University, Uppsala, Sweden

**Keywords:** Arabic/Swedish bilingual children, family language policy, heritage language education, home language maintenance, shared book reading, siblings

## Abstract

This paper investigates the language maintenance efforts of Arabic-heritage families whose preschool- and early primary school-age children are growing up bilingually with Arabic and Swedish. As a result of large-scale immigration to Sweden, Arabic has become by far the largest minority language, but very little is known about Arabic as a home language in this population. As part of a broader research project on child multilingualism, a questionnaire survey was administered to the parents of 100 Arabic/Swedish-speaking children. The resulting quantitative data on family language practices and beliefs were complemented by a smaller-scale follow-up interview study 2 years later. Family language practices targeted included parent-parent, parent-child and sibling interaction, language-fostering activities such as shared book reading, storytelling, and enrolment in home language education. Despite much diversity in family types concerning family constellations, parents' education, country of origin, Arabic language variety, and length of residence in Sweden, common traits emerge. Parents generally consider Arabic and Swedish to be equally important for their child to become proficient in. There is a strong focus on the transmission of Arabic in the home, and parents expect children to speak Arabic to them, though not all of them always do. In line with Swedish mainstream convention, most children are enrolled early in preschool. Parent-child interaction is reported to be mostly in Arabic, but in many homes, the agency of child and siblings leads to an increased use of Swedish, as does Swedish media consumption. In their home-language maintenance efforts, parents engage the help of extended family members, libraries, home-language teachers, and/or choose to enroll their child in a bilingual Arabic/Swedish preschool or a school with a particular profile that encourages Arabic. The families in the sample express little anxiety regarding their children's bilingualism and tend not to seek professional counsel in this matter. Swedish schools are reported to generally advise the parents to speak their native language as much as possible to their child. Whilst unusual from an international perspective, this finding is in line with minority-language maintenance advice previously documented for a different ethnolinguistic group in Sweden.

## 1 Introduction

This paper investigates the language practices and beliefs of families whose children grow up bilingually with Arabic and Swedish in Sweden. As a result of immigration, Arabic is by far the largest minority language spoken in Sweden now and has been so for some time [National Authority for Education (Skolverket), 2025]. Surprisingly though, not very much is known about the family language practices and beliefs of this population. Existing studies on family language policy and practices deal with other countries and settings (e.g. Lanza, 1997; Schwartz and Verschik, 2013; Schalley and Eisenchlas, 2020; Et-Bozkurt and Yagmur, 2022), while in the Swedish context, studies of multilingual family language practices have mainly focused on the Turkish-heritage population (e.g., Aktürk-Drake, 2017, 2018; Bohnacker, 2022, 2023).

Arabic is estimated to be spoken by 715,332 residents of Sweden as a native language, which corresponds to 7% of the 10.5 million national population. The vast majority of Arabic speakers in Sweden have their roots in Lebanon, Iraq and Syria due to different waves of mass immigration since the 1980s, mainly of refugees and family unification. Swedish authorities, such as the national statistics office, do not collect data on the number of speakers of a certain language, about home language use, or language skills. The above estimate is therefore based on census data for country of origin (Statistics Sweden, 2025), by combining the number of residents of Sweden born in an Arabic-speaking country (448,607) with the number of Sweden-born residents with one or two parents born in an Arabic-speaking country (266,665).^1^ The number remains an estimate since country of origin cannot straightforwardly be equated with language spoken, as there may be Arabic speakers in Sweden whose country of origin or that of their parents is not Arabic-speaking, and there may be other residents of Sweden who, despite having family roots in an Arabic-speaking country, have not grown up to speak Arabic themselves.

In Arabic-speaking countries, regional varieties (in Arabic ^*c*^ ā*miyya*) are used for daily, informal communication, while a standard variety (Modern Standard Arabic (MSA), in Arabic *fuṣḥa*) is used for formal communication and for official and liturgical purposes (e.g., in written discourse, education, religion, and media) (Suleiman, 2005). The regional varieties are often referred to as dialects or vernaculars; these differ from each other and from MSA in many respects concerning phonology, vocabulary and grammar (Abu-Rabia, 2000).^2^ In Sweden, the Arabic varieties most commonly spoken are Levantine (which includes Lebanese, Palestinian, Jordanian and Syrian (approximately 51%)) and Iraqi (approximately 33%) (Bohnacker, 2017; Statistics Sweden, 2025).

Children who grow up with Arabic do not acquire MSA as their native (or first) language (L1), rather, they are exposed to the regional variety in the home and acquire it as their L1; in Arab countries they later learn the standard variety at school. The situation is likely to be different for children growing up with Arabic in the diaspora, like Sweden. The parents may speak the same or different regional varieties, and the child will be exposed to these in the home, and not necessarily to additional Arabic elsewhere, but rather hear the societal language (Swedish) in (pre)school. If the child has Arabic-speaking peers, they may speak different regional varieties, and if the parents choose to enroll their child in Arabic home language education, the child may be taught to read and write in MSA by a teacher who may speak a different variety than the child's home variety (Bohnacker et al., 2025).

Enrolment in Arabic home language classes in Sweden is high (66%, National Authority for Education (Skolverket), 2025), which may indicate that parents generally set great store by their children becoming proficient in Arabic. International studies generally find that Arabic-speaking parents in immigration contexts value their language highly and are proud of it, wishing their children to learn it (e.g., Bentahila, 2011; Eid, 2019; Yousef and Taylor-Leech, 2018; Alraddadi, 2021). If this turns out to be the case for Sweden too, it could create good conditions for the maintenance of Arabic in a Swedish setting. However, positive attitudes are not in themselves enough for language maintenance (Hakuta and D'Andrea, 1992; Oller and Eilers, 2002; Spolsky, 2004; Pearson, 2007; Gafaranga, 2010), and the actual home language practices may be at odds with the parents' wish to pass on the language to their children.

Given the strong presence and dynamism of Arabic-speaking communities in the country, the scarcity of research on Arabic language maintenance in Sweden concerning intergenerational language transmission presents a significant knowledge gap. The current study investigates the practices of home language use and language beliefs of Arabic-heritage families in Sweden whose children are of preschool and early school age. The data presented are drawn from a broader research project and consist of 100 questionnaire survey responses from parents, and follow-up questionnaires and interviews conducted with 10 families. The data are discussed in light of an updated theoretical framework on family language policy and within the broader contextual background concerning people with a migratory background in Sweden and prevailing language ideologies.

The lack of any previous studies in this field makes it fitting to have a relatively detailed, empirical, descriptive approach. The following research questions are asked:

- What are the predominant family language practices (concerning parents, child, siblings, extended family, media, reading and other language-related activities, home language education, enrolment in different types of school)?
- Can the diversity in practices be explained by taking into account family background?
- Which attitudes and beliefs do the parents have toward multilingualism and home-language maintenance?
- How do family language practices change over time?
- Do the parents receive advice on their child's bilingualism, and how do they act on it?

The paper is structured as follows. Section 2 presents relevant frameworks for intergenerational language transmission and family language policy (including the agency of parents, children, teachers, and the wider socio-political context). Section 3 outlines the method of the study, including the sociolinguistic background of the participants. Section 4 reports on the empirical findings from the parental questionnaire survey and the follow-up interviews, linking quantitative with qualitative results. Finally, Section 5 discusses these family language practices and beliefs in light of theoretical approaches to minority-language maintenance and family language policy, against the backdrop of multilingual language ideologies in Sweden.

## 2 Background and theoretical framework

### 2.1 Intergenerational language transmission

Children acquire language in social networks. For young children, the home and in particular the interactions between parents and child (or other primary caregivers and child) are generally considered to be the pivot around which the intergenerational passing on of minority languages revolves (e.g., Fishman, 1972, 1991; Lanza, 1997; Pearson, 2007).

Classic sociological and sociolinguistic theorizing has often approached language transmission, maintenance and shift from a *generational* perspective (Veltman, 1988; Fishman, 1972, 1991).^3^ A popularized version of such approaches is the three-generation model: The first generation of immigrants (Generation 1) speak their native language (L1) and retain it as a home/minority language in the new setting; they also learn the new majority/societal language as a second language (L2), though often imperfectly so.^4^ They transmit their native language to their children (Generation 2), who grow up bilingually with both the heritage language and the societal language. Generation 2 speakers are assumed to become dominant in the societal language and not to always pass on the heritage language to their children. Members of Generation 3 won't speak the heritage language fluently but become (largely) monolingual speakers of the societal language, even though the linguistic and cultural heritage may be important for them for reasons of identity.

Whilst such an assimilationist three-generation model may well capture patterns of language transmission and shift in some families and contexts, empirical studies over the past decades have shown that it does not capture them in others. The vitality of minority languages may vary widely between heritage languages and across contexts (Giles et al., 1977; Yagmur and van de Vijver, 2022). Influenced by a multitude of factors, processes of acculturation and language shift to the majority language can be slower, stretching over more than three generations—or faster (Hakuta and D'Andrea, 1992; Pearson, 2007; Schwartz, 2010). Moreover, family members cannot always be labeled as Generation 1, 2 or 3, but might be somewhere in between: Generation 1.5 immigrated as children and were schooled in the new societal language, which may promote language shift (Veltman, 1988; Hakuta and D'Andrea, 1992; Rumbaut, 2004; Pearson, 2007). Generation 2.5 might be born in the new country with one second-generation parent but the other parent being a first-generation newcomer, which revitalizes the use of the heritage language in the family.

### 2.2 Family language policy and child agency

Other sociolinguistic approaches have put forward the *agency* of family members and *family language policy* (Luykx, 2005; King et al., 2008) as explanatory concepts for home language use and maintenance.^5^ To begin with, family language policy was narrowly defined as “explicit and overt planning in relation to language use within the home among family members” (King et al., 2008: p. 907), i.e., deliberate planning, but was soon broadened to also include non-deliberately evolving language practices (Lanza and Lomeu Gomes, 2020). It is from the language practices that the “real” family language policy emerges.

(Spolsky 2004) proposed a tripartite language policy model for speech communities (e.g., classrooms, schools, institutions, nation states), comprising (i) practices, (ii) beliefs/ideology, and (iii) management/intervention/planning (Spolsky, 2004: p. 5). Applied to the domain of the minority-language family (Spolsky, 2012), family language policy would thus comprise (i) family *language practices*, (ii) *language beliefs*, i.e., beliefs about language and language use, and (iii) *language management*, i.e., specific decisions and efforts undertaken by persons of authority (often the parents) to modify or influence language practices. Such management efforts are by no means guaranteed to have the intended effect though. Also, language beliefs and attitudes^6^ do not only shape language practices but may also be shaped by them. Indeed, family language policy can be dynamic and subject to change.

In the field of family language policy, child agency has only gained traction in recent years (Schwartz, 2010; Smith-Christmas, 2020). Agency refers to the ability to make choices and initiate change. Early landmark studies by (Döpke 1992) and (Lanza 1997) analyzed parent-child interactions in German/English-speaking and Norwegian/English-speaking families, showing how young children may strongly influence language use in the home, but also how parents can steer this, by employing certain discourse strategies that are oriented toward minority-language maintenance. Such strategies can be ranked according to the degree to which they promote the use of the minority language by the bilingual child in conversations (or not). More recent empirical studies have shown how the child can exercise their own agency (e.g., Gafaranga, 2010; Fogle and King, 2013), as can siblings (Barron-Hauwaert, 2011; Bridges and Hoff, 2014; Kheirkhah and Cekaite, 2018; Sorenson Duncan and Paradis, 2020). This is because children often bring the majority language into the home, speak it, and invite other family members to speak it with them, which can promote a general shift away from the minority language. Here, children may come to challenge or ignore aspects of the parents' desired home language use (Luykx, 2005). This is an example of a family language practice that often simply “happens” or evolves. Other language practices in the family may be due to explicit and deliberate language management efforts by the parents, such as measures taken to actively boost input and/or interaction in a particular language in or outside the home, e.g., parents insisting that the minority language be spoken at the family dinner table, joint visits to the library to borrow home-language children's books, hiring a language-specific childminder or having L1 grandparents mind the child, singing, telling or reading bedtime stories in a particular language, or enrolling one's child in home-language education classes or in a bilingual (pre)school instead of the monolingual mainstream one. Such deliberate practices of controlling the external sociolinguistic environment of the child and/or controlling the language environment at home are considered to be clear manifestations of family language policy (e.g., Luykx, 2005; King et al., 2008; Schwartz, 2010; Curdt-Christiansen, 2018), though many researchers also subsume non-deliberately evolving family language practices under this term (Lanza and Lomeu Gomes, 2020).

A case in point is when the expanding social circle (e.g., friends, peers, school, digital communication) of children from minority-language families increasingly impacts whether and how the minority language continues to be practiced and developed. This influence may be at odds with the family language policy originally advocated by the parents. Children are quick to perceive and integrate the image that peers and teachers may have of them and their home languages, and adjust their language use accordingly (Mary and Young, 2020).

### 2.3 Teachers, school and the wider sociopolitical context

Teachers, preschool staff and child healthcare professionals may also exert considerable agency when counseling parents directly on their child's bilingual language development. These persons of authority may proffer advice that aligns or clashes with the parents' language practices and beliefs. Parents often act on such advice, since they generally want their children to do well in school; teacher agency may thus boost or curtail the child's future development of the minority language. Studies from countries such as Belgium, Germany and the Netherlands (Montanari, 2017; Pulinx et al., 2017; Bezçioglu-Göktolga and Yagmur, 2018) have found that (pre)school teachers routinely counsel against the use of the minority language in the home and instead advise minority-language parents to speak and read the majority (societal) language with the child, a recommendation for which there is no research basis.^7^ Such advice stems from the belief that increased contact with the minority language will prevent sufficient contact with the majority language. As pointed out by (Mary and Young 2020: p. 447), by giving such advice “the teacher not only shifts the responsibility for school language development from the school to the home, but also endorses the idea that parents have to make a ‘choice' between using the home language with their child or prioritizing their child's future.” Again, this idea of a forced choice does not have any basis in research.

A recent study from Sweden (Bohnacker, 2023) has found a different pattern though: Turkish-heritage parents of preschool- and primary-school-age children in Sweden reported throughout that they had been advised by teachers, educational experts and speech-language therapists that they should do their utmost for their child to learn Turkish really well. They should therefore strive to speak as much Turkish as possible with the child and maximize input to Turkish in the home. The rationale behind this advice was that a well-developed minority language would help the child to learn other languages more easily, including the majority language Swedish. In essence, the rationale is equivalent to the interdependence hypothesis, according to which proficiency in the first language (L1) will strengthen and speed up the acquisition of the second language (L2) and other subsequent languages (e.g., Skutnabb-Kangas and Toukomaa, 1976; Cummins, 1979, 1991; Thomas and Collier, 1997). We are not aware of any research that investigates the kind of language advice school teachers provide to other minority-language groups, such as Arabic-heritage families in Sweden.

Family language policy may also be shaped and altered by the wider sociopolitical context and the dominant language ideology of the receiving society toward multilingualism and minority languages. Sweden is generally known for its multiculturally oriented politics and longstanding state-level support for home-language education (Aktürk-Drake, 2017, 2018; European Commission Directorate-General for Communication, 2018; Salö et al., 2018; Migration Integration Policy Index (MIPEX), 2020). The 2009 Language Act (*Språklagen*) states that Swedish is the principal language of the country, but the same piece of legislation also enshrines minority language rights (Language Act (Språklagen), 2009). The 2010 Education Act (*Skollagen*) gives children the right to minority language education nationwide (*modersmålsundervisning* “mother tongue instruction”) (Education Act (Skollagen), 2010). What sets Sweden apart from many other countries is that home language education is funded by the government, offered as an elective subject within the regular school system, and that preschool and school-age children who grow up with a home language other than the majority language Swedish are entitled to this by law (For detailed discussions of the Swedish home-language education system past and present, see e.g., Hyltenstam and Milani, 2012; SOU 2019:18, 2019; Bohnacker, 2023).

In the currently changing Swedish sociopolitical climate, mismatches emerge between the official state-level language policy promoting minority-language maintenance and multilingualism, the municipalities' implementation of this policy, and increasingly louder voices in the public domain. Both in parliament and in the media, Swedish right-wing parties are calling for cuts or the complete abolishment of home language education, which could signal a change in societal attitudes toward multilingualism in minority-language children (Bohnacker et al., 2025). In media campaigns, the political far-right appear to have singled out Arabic as a particularly undesirable heritage language, which may not have gone unnoticed by Arabic-heritage families in Sweden.^8^

As mentioned above, language practices and beliefs in Arabic-heritage families in Sweden are an under-researched area. There is one unpublished MA thesis (Attaallah, 2020) where 5 Arabic-speaking immigrant parents were interviewed about home language maintenance when bringing up children in Sweden. These parents came from different backgrounds and reported different language practices in the home, but all valued Arabic highly and wanted their children to become proficient in it. They emphasized that parents should communicate this wish explicitly and continuously to their children. Some parents viewed the Arabic language as integral to their sociocultural and/or religious identity and wanted their children to do the same. Others were of the opinion that their children should learn Arabic alongside Swedish so that they could be free movers between different cultures, countries and communities. The small number of families in the study makes generalizations difficult. We are not aware of any larger-scale studies, other than our own work (Bohnacker et al., 2025).^9^

## 3 The present study: materials and methods

### 3.1 The project

The research reported in this study is part of a broader child multilingualism research project (BiLI-TAS) directed by the first author, which investigates the language development of preschool and primary school children growing up in Sweden with two or more languages. The larger project included a sample of 100 Arabic/Swedish-speaking children (age 4–7) and their families, who are the participants in the present study. A subgroup of these children and their families were seen again some years later, to gain a longitudinal perspective. The main objective of the larger project was to measure and compare the children's language skills in their home language (e.g., the Arabic vernacular) and in the majority language Swedish. The children carried out a range of tasks measuring their expressive and receptive language skills concerning vocabulary, narrative and phonological processing in both languages. These are not in focus in the present paper and will therefore not be reported here (for results, see Öberg, 2020; Bohnacker et al., 2021a,b; Haddad, 2022; Öberg and Bohnacker, 2022). In order to better explain the children's language results, we systematically explored the language and social backgrounds of the families. This was done through questionnaire surveys and interviews. The questionnaire surveys and interviews with the parents also provide rich data concerning the families' language practices, policies and beliefs, which are the focus of the present study.

### 3.2 Data collection, processing, and analysis

One hundred Arabic-speaking families in Sweden with children in the age range of 4;0 to 7;11 years were recruited by contacting a large number of (pre)schools in urban regions of eastern central Sweden, as well as cultural associations and congregations that organized activities for Arabic-speaking children. Some families were recruited through personal contacts of Arabic-speaking research assistants and through bilingual events at community centers. Randomized sampling from the national population register was not feasible, as no statistics are kept on whether a resident of Sweden speaks Arabic or not. Instead we used non-probability judgmental/purposive sampling, also called criterion sampling (Dörnyei, 2007: p. 126–128; Lanza, 2008: p. 82–85). The criteria to be fulfilled for inclusion in the sample were: child aged 4–7 years; no known hearing, language or neuropsychiatric disorders; Arabic spoken in the home; Arabic and Swedish spoken by the child; child resident in eastern central Sweden; willingness to participate. We aimed to recruit children who were active bilinguals, i.e., who could speak both languages (Arabic and Swedish) at least to some degree, since the project involved expressive language tasks in both languages for the children.^10^ The children came from 53 different (pre)schools. We targeted urban regions of eastern central Sweden, including the metropolitan region of greater Stockholm and neighboring cities, because a large share of the Arabic-speaking population in Sweden is resident there (Bohnacker, 2017) and there is a good mix of inner cities and suburbs oriented toward the industrial, service and education sectors. In addition, recruitment and data collection there was logistically viable.

Families received oral and written information about the project in both Arabic and Swedish. Those parents who agreed to participate provided informed written consent for their and their child's participation. Children assented orally. Participation could be discontinued at any time. Procedures were put in place to preserve the confidentiality of participants and to ensure that the research was done in an ethical way (Haddad, 2022). Anonymity of the families was ensured by assigning individual codes. The study has been carried out in accordance with the local university ethical code of conduct and Swedish regulations concerning data protection.

The parents of the children filled in an extensive questionnaire (five pages, paper-and-pencil) in the language of their choice, Arabic or Swedish, and those who preferred to respond orally were interviewed in Arabic by trained researchers who filled in the questionnaire with them.^11^ The questions in the questionnaire were developed by the BiLI-TAS project team for several languages and piloted with Arabic-speaking parents and members of the Arabic community in Sweden. The questionnaire was inspired by international questionnaires such as the PABIQ (Parents of Bilingual Children Questionnaire; Tuller, 2015), and questionnaires used by speech-language therapists in Sweden. There were 36 questionnaire items (some yes/no, some estimation scales, and some open-ended questions) targeting the child's age at first regular exposure to Arabic vs. Swedish, (pre)school attendance and school language(s), birth order, the child's early language development, the parents' background with regard to language, education and occupation, parents' length of residence in Sweden, family language use in the home, including the parents' language(s) spoken with each other and to the child, the language used by the child to communicate with their parents and siblings, language use between siblings, language use with extended family etc. Parents also estimated the proportion of daily input to the child for each language and the frequency and extent of language-related activities in Arabic and Swedish in and outside the home, such as media consumption, shared book reading, singing and storytelling, as well as home language education. Parents were also asked which language(s) they considered most important for their child to become proficient in, whether they had ever felt any anxiety about the child's language development and why, and what their experience had been when seeking help or advice in such cases (The questionnaire is available as Supplementary material).

Questionnaire data was available for all 100 children, though in some cases, parents omitted to answer certain questions, and for one child most questions were left blank. Parental responses were anonymised, coded, entered into spreadsheets and analyzed/processed quantitatively in Excel and SPSS. They form the database for the questionnaire survey results, which will be referred to as the *large sample* or the *large-scale survey*.

Approximately 2 years after participation in the original study, a subgroup of the participants were seen again, to provide insights on the longitudinal development from age 4 to age 6. We recontacted all 22 families whose child had been 4 years old in the cross-sectional study; however due to contact and travel restrictions during the COVID pandemic, we were only able to collect data from 10 of these children and their families (45%).^12^ As described in Section 3.3, the backgrounds of the 10 children and their families in the longitudinal follow-up largely reflect the profiles of the 100 children in the original sample. The children carried out the same language tasks as 2 years earlier (not reported here, see Haddad, 2022), and we also briefly interviewed them about their language use with parents and sibling(s), with other relatives and friends, and whether they had any (pre)school teachers and/or classmates that they spoke Arabic with (15 questions).

The bulk of the information we gathered about family language use in the longitudinal follow-up came from the parents though, who filled in a questionnaire (in Arabic or Swedish) and were interviewed by the second author, a native speaker of Arabic. The questionnaire was a shortened version of the original questionnaire (17 questions about language use and attitudes now that their child was 2 years older). The questionnaire responses were coded, entered into spreadsheets and analyzed quantitatively. The interview was carried out as a structured interview with the mother of the child, either during a home visit, or in a couple of cases via telephone, when contact and travel restrictions made in-person meetings difficult during the COVID pandemic. The interviews focused on the family's language practices and attitudes and which aspects of language use might have changed over time. We also asked about Arabic-speaking teaching staff at the child's (pre)school, language contact with grandparents and other relatives, library visits and reading habits, interaction strategies, whether the parents ever consulted anyone about their child's language development and bilingualism, and what kind of advice they would give to parents of other bilingual children to learn Arabic in Sweden. The parental interview responses were transcribed, anonymised, entered into spreadsheets and analyzed thematically by hand. They form the database for the *longitudinal follow-up*.^13^

Some preliminary observations from the large sample and the longitudinal follow-up were reported in (Haddad 2022). The data have since been cross-checked, cleaned and analyzed further by the first author and PI of the BiLI-TAS project. The questionnaire and interview data are reported here in detail for the first time and discussed in relation to family language policy.

In order to make sense of the diversity in family language policies, we explored family background in relation to language practices, and language practices in relation to language attitudes and beliefs. We adopted a mixed-methods approach that involved “the collection and analysis of both quantitative and qualitative data in a single study” (Dörnyei, 2007: p. 163), the rationale being that this allows for triangulation and may improve the validity of the findings (Dörnyei, 2007). The quantitative data (from 100 parental questionnaires) reveal a generalisable picture of family language practices and attitudes, whilst the qualitative data from the interviews provide deeper insights into these practices, attitudes and beliefs and the motivations behind them.

### 3.3 Background of the participants

The families of the 100 Arabic/Swedish-speaking children that participated in the cross-sectional study lived in urban regions in eastern central Sweden. According to parental report, the children had no known hearing, language or neuropsychiatric disorders at the time. The 100 children, 50 girls and 50 boys, broke down into 22 4-year-olds, 25 5-year-olds, 29 6-year-olds, and 24 7-year-olds.

Nearly all children (95/100) grew up in two-parent households, the remainder lived with single parents or alternated between two different households. Nearly all children (94/100) had siblings. Thirty-nine children were first-borns (and thus did not have an older sibling to interact with), whereas 61 children had at least one older sibling. Roughly half the children (55/100) were born in Sweden, whereas 44 were born in an Arabic-speaking country and had come to Sweden as toddlers. One child was born in an English-speaking country.

Table 1 provides a generational breakdown of the participants by their history of immigration. Most children (88%) belonged to Generation 1.5, i.e., they immigrated as children (recall Section 2) or to Generation 2, i.e., they were born in Sweden but both their parents had been raised in an Arabic-speaking country and had immigrated to Sweden as adults. The remaining children (12%) had one parent who was raised in Sweden: In one family, one parent had been born and raised in Sweden, in 11 families one parent had come to Sweden as a child and had (at least partially) grown up here, but there were no families with two parents who had grown up in Sweden. Parental length of residence in Sweden varied from only 10 months to 40 years, with an average length of 9.7 years.

**Table 1:** Breakdown of the children by their and their parents' history of immigration.

**Generation**	**Entire sample *N* = 100**	**Longitudinal sub-sample *N* = 10**
G1.5 (child born outside Sweden, parents raised in Arabic-speaking country)	45	2
G2 (child born in Sweden, parents raised in Arabic-speaking country)	43	8
G2.5 (child born in Sweden, 1 parent raised in Sweden)	12	0
G3 (child has two Sweden-raised parents)	0	0

The families' socioeconomic status varied greatly concerning parents' occupations and formal education levels, which spanned from only 3 years of primary school to doctoral degrees. Many parents had completed upper secondary school, and about half had at least some tertiary (college) education. Many had originally trained in white-collar professions but were now employed in other, less skilled, jobs in Sweden, often in the service, transport, and crafts sectors, or attended vocational training and/or Swedish language classes. Roughly 25% were currently homemakers or unemployed. Regardless of employment status and family income though, all children had access to public daycare and schooling.

Nearly all parents considered Arabic to be their first/native language (L1) and to be fully fluent in it; none reported Swedish as their L1. One parent had a different language as their L1 but conversed in Arabic. The families spoke different Arabic varieties, roughly reflecting the linguistic diversity of the Arabic-speaking population resident in Sweden, where varieties from the Middle East, in particular, the Levant (i.e., eastern Mediterranean, e.g., Syrian, Palestinian, Lebanese) and Iraq predominate.

Parents were also asked to rate their knowledge of Swedish. The answers varied greatly. Fourteen percent rated their Swedish as fluent; these were mainly parents who had lived in Sweden for more than 12 years, often 20–30 years, and some had also grown up in Sweden. Thirty-five percent parents rated their Swedish as intermediate-level and 20% as basic or non-existent. The remaining parents (31%) did not provide information regarding their knowledge of Swedish. Leaving this question blank may indicate low proficiency in Swedish.

Almost all children (98/100) had been continually exposed to Arabic from birth, which is to be expected considering the parents' language background. For one child, Arabic exposure was reported to have started shortly after birth, and for one child such information was missing. By exposure to “Arabic” we mean exposure to colloquial, regional varieties (^*c*^ ā*miyya*), as these are the ones spoken in the home (and not MSA/*fuṣḥa*). All children were reported to be exposed to a regional variety of Arabic in the home. Only one family reported that they sometimes also spoke MSA to the child, to teach the child the standard variety. Twenty-five percent of the children were reported to be exposed to more than one regional variety in the home, e.g., via television, gaming, or the parents speaking different regional varieties. According to parental report, the children spoke mainly Syrian and Palestinian varieties of Arabic (43 and 26 children, respectively), followed by Iraqi (*N* = 17), Lebanese (*N* = 9), and Egyptian (*N* = 4).

Age of onset of Swedish varied for the children from before age 1 to shortly after age 6. Only 6 children had been regularly exposed to Swedish during their first year of life. For half the children (52/100), regular exposure to Swedish had started after age 3;0. Children with later onsets for Swedish had often been born abroad and had immigrated to Sweden relatively recently with their families. Most children were characterized by their parents as bilingual (Arabic, Swedish), but two children grew up in homes where in addition a third language was spoken on a regular basis (English, Kurdish).

The backgrounds of the 10 children and their families in the *longitudinal follow-up* were similar to those of the larger sample. All parents reported Arabic as their native (L1) language, they had been raised in an Arabic-speaking country and immigrated to Sweden as adults. Parental education levels ranged from primary education to postgraduate degrees. For all children, age of onset for Arabic was at birth, while age of onset for Swedish again showed more variation (from before age 1 to between age 3–4). A higher proportion of children (8/10) in the longitudinal follow-up than in the cross-sectional sample was born in Sweden (see Table 1, right-hand column). Again, Syrian and Palestinian varieties of Arabic predominated.

## 4 Results

### 4.1 Parents' language use and parent-child interaction

The language the parents speak with each other at home gives an idea of what language the child is exposed to on a daily basis, even if this language is not directly addressed to the child. In the *large-scale survey*, the vast majority of the parents (90%) reported that they spoke solely Arabic with each other.^14^ No household reported that the parents predominantly spoke Swedish to each other. The parents of three children reported that they spoke both Arabic and Swedish to each other. These parents had typically had lived in Sweden for many years and one of them explicitly commented: “I came to Sweden when I was 10 years old and I can express myself best in Swedish” (parent of BiAra5-06). For the remaining households, no information was available regarding parental language use with each other.

Let us now consider parent-child interaction. The language the parents speak to the child on a daily basis constitutes a direct language input source. The language chosen for interaction with the child is also a reflection of family language policy, whether it is consciously planned or not. In our study, the parents were asked to specify on a five-point scale whether they spoke with their child “almost only Arabic,” “mostly Arabic,” “even” (i.e., fifty-fifty), “mostly Swedish,” “almost only Swedish”; they could also fill in “other” if their language use did not match that scale. For the large majority of children (79/100), both parents spoke only or mostly Arabic with their child (see Table 2). The only parent who reported to speak mostly Swedish with their child was the same parent mentioned above who had grown up in Sweden and also preferred to speak Swedish with their spouse. For 19 children, the parents spoke both Arabic and Swedish to the child, to varying degrees (for details, see Table 2, left column). The parents of two children reported “other”, involving a third language (Kurdish, English). Among the (few) parents who reported to speak at least 50% Swedish to their child, longer residency in Sweden tended to be common, often 12–30 years (Conversely however, it was not the case that all parents who had lived in Sweden for such a long time years reported speaking a lot of Swedish to their child).

**Table 2:** Language use between parents and child, entire sample (100 children), questionnaire responses.

**Parents speak to child**		**Child speaks to parents**	
Both parents mostly Arabic	79	To both parents mostly Arabic	68
Both parents mostly Swedish	0	To both parents mostly Swedish	8
1 parent mostly Arabic, 1 parent 50/50 or mostly Swedish	9	To 1 parent mostly Arabic, to 1 parent 50/50 or mostly Swedish	8
1 parent mostly Swedish, 1 parent 50/50	0	To 1 parent mostly Swedish, to 1 parent 50/50	1
Both parents 50/50	9	To both parents 50/50	13
Other	2	Other	1
Missing information	1	Missing information	1
Total	100	Total	100

“50/50” indicates roughly equal amounts of Arabic and Swedish.

The parents were also asked to report which language(s) the child speaks to them. Table 2 (right-hand column) shows the child's language use with the parents. The majority of the children (68/100) were reported to speak mostly Arabic with their parents. The parents of 8 children, however, reported that their child spoke mostly Swedish to both of them. This pattern differs from parent-to-child language use, where no household indicated that both parents spoke mostly Swedish to the child. The parents of 22 children reported that their child spoke both Arabic and Swedish to them, to varying degrees.^15^ One child was reported to speak mainly Arabic to one parent and Kurdish and Swedish to the other parent (this was the parent whose L1 was Kurdish). For one child, information regarding language use with the parents was missing.

One might expect that children growing up with parents who themselves have grown up in Sweden speak more Swedish with their parents than those whose parents have come to Sweden as adults. However, this does not appear to be the case in our sample. Only two of the 12 children with a Sweden-raised parent were reported to speak a lot of Swedish to their parents (50/50 or mostly Swedish), but 29 other children (without any Sweden-raised parent) were also reported to speak a lot of Swedish to their parents (50/50 or mostly Swedish). This suggests that a purely generational approach to home language use is too simplistic (Fishman, 1972, 1991; Pearson, 2007) and that other social and affective factors influence children's language use with their parents in the home. These are investigated in the following sections.

In the *longitudinal follow-up*, all parents reported that they solely spoke Arabic to each other. The majority of the parents spoke only or mostly Arabic to the child, while others reported that they spoke both Arabic and Swedish, with some shifts toward more Swedish, and some shifts toward more Arabic (see Table 4). Concerning child-to-parent speech, all but one child (BiAra4-02, who mostly spoke Swedish to their parents) were reported to speak mostly Arabic or a mixture of Arabic and Swedish, with little change over time (see Table 4). These data are based on a comparison of the parents' answers in the questionnaires at the two data collection times (age 4 and age 6); the interviews generally confirmed this pattern, with some exceptions: In the interview, the parent of BiAra4-14 reported that the child nowadays mostly spoke Swedish to the parents, even when spoken to in Arabic. The interviews also revealed that some parents had started to speak more Swedish to their child (BiAra4-02, BiAra4-06) because they had experienced that s/he had difficulty in understanding everything that was being said in Arabic and/or because the child preferred Swedish. Other parents reported that they purposely spoke more Arabic to the child now than 2 years earlier, in order to maintain the home language and because they foresaw their child needing strong Arabic language skills in the future (BiAra4-16, BiAra4-18). In six families, the parents reported no change in parent-child language use over the past 2 years.

In the interviews, we also asked the parents about interaction strategies (Lanza, 1997), for instance, whether it happened that the parent spoke Arabic and the child would respond in Swedish, and if so, what the parent would do. For 5 children, the parents said that this situation did not occur or very rarely because the child always spoke Arabic with them (these families had not been in Sweden for very long). The other parents (with varied residence lengths) acknowledged that such situations did occur, but they handled it in different ways. Some answered that they insisted on continuing in Arabic, that they rephrased or explained in Arabic, that they reminded the child that they did not understand Swedish and told the child to speak Arabic. Several parents pointed out that these strategies did not always work. For instance, one mother said: “I try to ‘force' her to speak in Arabic, by telling her (in Swedish) that I don't understand Swedish. I encourage her to speak Arabic with me.” Another mother said: “I try to correct her and make her speak in Arabic, if she does not understand, then I try to explain to her in Swedish.” Two parents reported that their child at times would refuse to speak Arabic to them, that they would get exasperated and then continue in Swedish. We see a strong sense of child agency here. Still, for all but one family, parental language use with the child appears to remain predominantly in Arabic.

### 4.2 Language use with siblings

Compared to parent-parent and parent-child interaction (which, as we have seen, was largely in Arabic), language use between siblings was different. Parents were asked to choose one option out of five: “no sibling,” “mostly Arabic,” “both Arabic and Swedish,” “mostly Swedish,” or “other language.” As shown in Table 3, in the *large sample*, 39% (39/100) children were reported to communicate mostly in Arabic with their siblings (vs. 68% mostly in Arabic with their parents, recall Table 2). Eighteen percent were reported to speak mostly Swedish with their siblings (recall that only 8% did this with their parents). Thirty-six percent spoke both Swedish and Arabic with their siblings. In four cases, the parents also reported “other” language use between siblings, namely the use of English in addition to Swedish and/or Arabic.^16^ Six children did not have siblings; and for one child, sibling information was missing.

**Table 3:** Language use between child and siblings, questionnaire responses.

**Child speaks to sibling(s)**	**Entire sample *N* = 100**	**Longitudinal sub-sample *N* = 10**
Mostly Arabic	39	6
Arabic and Swedish	36	3
Mostly Swedish	18	2
Missing information	1	0
No siblings	6	0

We were interested to find out whether the 18 children who spoke mostly Swedish with their siblings had any other traits in common. There was no observable pattern regarding birth order or the number of siblings. However, two thirds of the children who spoke mostly Swedish with their siblings (13/18) were also reported to speak a lot of Swedish to their parents (mostly Swedish or 50/50). A few (4/18) had a parent who had grown up in Sweden themselves, though most of them did not. Interestingly, nearly all of the children who mostly spoke Swedish with their siblings (15/18) were born in Sweden. The reverse did not hold though (i.e., there were other Sweden-born children who were not reported to mostly speak Swedish with their siblings, but Arabic or a mix). We could thus not see any generational patterns here.

In the *longitudinal follow-up*, we noted that the parents' written follow-up questionnaire responses concerning language use between siblings did not always match the information provided orally during the interview; several families reported that the child now codeswitched more into Swedish when communicating with siblings. Four children were reported to continue to speak mostly Arabic with their sibling(s) (see Table 4, rightmost column). The other six were reported to either speak a mixture of Arabic and Swedish or mostly Swedish with their sibling(s).

**Table 4:** Language use between parents, parents and child, child and siblings, questionnaire responses in the longitudinal follow-up (10 children, age 4 vs. age 6).

**Child code**	**Age**	**Parents speak to each other**	**Parents speak to child**	**Child speaks to parents**	**Child speaks to sibling(s)**
BiAra4-02	At age 4	Arabic	Both only Arabic	To both mostly Swedish	Arabic and Swedish
	At age 6	Arabic	Both Arabic and Swedish	To both Arabic and Swedish^*^	Arabic and Swedish^*^
BiAra4-05	At age 4	Single parent	Mostly Arabic	Mostly Arabic	Mostly Arabic
	At age 6		Mostly Arabic	Mostly Arabic	Arabic and Swedish^*^
BiAra4-06	At age 4	Arabic	Both only Arabic	To both only Arabic	Arabic and Swedish
	At age 6	Arabic	1 only Arabic, 1 mostly Arabic	To both Arabic and Swedish^*^	Arabic and Swedish^*^
BiAra4-08	At age 4	Arabic	Both only Arabic	To both only Arabic	Mostly Arabic
	At age 6	Arabic	Both only Arabic	To both only Arabic	Mostly Arabic
BiAra4-13	At age 4	Arabic	Both only Arabic	To both mostly Arabic	Mostly Arabic
	At age 6	Arabic	1 only Arabic, 1 mostly Arabic	To both mostly Arabic	Mostly Arabic^*^
BiAra4-14	At age 4	Arabic	1 only Arabic, 1 mostly Arabic	To 1 only Arabic, to 1 Arabic and Swedish	Mostly Swedish
	At age 6	Arabic	1 only Arabic, 1 Arabic and Swedish	To 1 mostly Arabic, to 1 Arabic and Swedish^*^	Mostly Swedish
BiAra4-15	At age 4	Arabic	Both Arabic and Swedish	To both Arabic and Swedish	Mostly Swedish
	At age 6	Arabic	Both mostly Arabic	To both mostly Arabic	Mostly Arabic^*^
BiAra4-16	At age 4	Arabic	Both Arabic and Swedish	To both mostly Arabic	Mostly Arabic
	At age 6	Arabic	Both only Arabic	To both only Arabic	Mostly Arabic^*^
BiAra4-18	At age 4	Arabic	Both Arabic and Swedish	Mostly Arabic	Mostly Arabic
	At age 6	Arabic	Both only Arabic	To both only Arabic	Mostly Arabic
BiAra4-24	At age 4	Arabic	Both mostly Arabic	To both only Arabic	Mostly Arabic
	At age 6	Arabic	Both only Arabic	To both only Arabic	Mostly Arabic

^*^During the interview, parents adjusted this estimate toward more Swedish.

For most of the ten children, it became evident during the interview that language use between siblings in fact had shifted toward more Swedish (which could mean that the child continued to speak Arabic but now interspersed with a little Swedish, or that the child had shifted from mostly Arabic to a combination of Arabic and Swedish or to mostly Swedish). In fact, the parents of five children pointed out that their children predominantly spoke Swedish with their sibling(s) at home (BiAra4-02, BiAra4-05, BiAra4-06, BiAra4-14, BiAra4-15). These siblings were all older than the child. Some parents mentioned that they strongly encouraged their offspring to speak Arabic with each other, but that the children often refused to do so and continued in Swedish. For instance, one mother said “With her older sister she speaks in Swedish, but I tell them to speak more Arabic. But when they are together, they just speak Swedish.” We see examples of strong child agency here. In three cases (BiAra4-02, BiAra4-05, BiAra4-16), the follow-up interviews moreover revealed that the children were also increasingly speaking some English with their sibling(s). According to the parents, children showed an interest in speaking English since they had started to learn it at school and/or were being more exposed to it while using electronic devices at home.

### 4.3 Language use with grandparents and extended family

We also asked about *grandparents* and other relatives as additional language input providers. In the literature, relatives, especially those of the older generation, are generally regarded as important for the transmission and upkeep of the heritage language and culture. In the *large sample*, a large majority of the children (78/100) was reported to hear Arabic from extended family. For 21 children this was not the case; we could not determine any commonalities concerning the background of these families. In 5 cases no information was available.^17^

In the *longitudinal follow-up*, we explored the issue of language use with extended family further. During the interviews, it emerged that all 10 children spoke Arabic with their grandparents, and some also with aunts, uncles, cousins and other relatives (this had not been queried in the questionnaire). Relatives were contacted via telephone or online video/audio call, since they often did not live in Sweden. With major recent advances in digital telecommunication technology, grandparents and other relatives can now be co-present in the lives of families to a greater extent than in the past. Several parents stressed that being able to stay in contact with their extended family was their main motivation to make the child learn Arabic. It seems that when children interact with relatives, they are more or less forced to use the heritage language since the relatives do not understand Swedish. For instance, one mother said “He speaks mostly in Arabic with those that are in [country of origin] since he has no other choice with his grandparents, but some relatives are in Sweden so they switch to Swedish.” The informal interviews with the children confirmed these insights, though another child mentioned that s/he also spoke Swedish with relatives who lived in Sweden. In one family, the child had begun to communicate in English (rather than Arabic) with non-Swedish-speaking cousins, uncles and aunts. Nearly all of the children reported that they sometimes went abroad with their parents to meet Arabic-speaking friends and relatives, and/or that such relatives had been to visit them in Sweden and that they then communicate in Arabic.

### 4.4 Language-related home activities

Joint activities such as storytelling, singing, watching films or reading books together in a particular language can also reflect family language policy, irrespective of whether the language used for the activity has been consciously chosen or not. As is well-known, such fun activities together nurture socioemotional bonds between family members and can build language and literacy skills. To gain further insights into such home activities, we asked parents how often they had performed the following with their child during the past month: telling stories, reading books (or looking at picture books) together, singing and/or listening to songs, watching TV/videos or doing computer activities together (Table 5). For each language (Arabic and Swedish), parents specified each type of activity on a 4-point scale (“almost every day,” “once/twice a week,” “twice a month,” or “(almost) never”). Although it is not possible to measure the richness of the language that is being provided in the books the children read or the songs they hear, such home activities ensure a certain amount of language input.

**Table 5:** Language-related activities in Arabic and Swedish in the home, entire sample (100 children).

**Arabic**	**Never**	**Twice a month**	**Once/twice a week**	**Almost every day**	**Missing information**
Telling stories	7	17	42	14	20
Shared book reading	12	13	39	14	22
Singing/listening to songs	10	12	22	37	19
Watching TV/videos	6	6	15	62	11
**Swedish**	**Never**	**Twice a month**	**Once/twice a week**	**Almost every day**	**Missing information**
Telling stories	26	16	20	16	22
Shared book reading	19	15	29	20	17
Singing/listening to songs	15	14	22	27	22
Watching TV/videos	15	7	12	53	13

The joint activity reported most frequently was watching videos/films (on television, computers or other electronic devices); and for a large majority of children, parents reported doing this together with their child every day or at least once a week, in Arabic (77/100), and in Swedish (65/100). Slightly more than half the families also reported that they told the child stories in Arabic (56/100), read books in Arabic for them (53/100) or sang Arabic songs with the child (59/100) at least once a week. On the whole, such joint activities seemed to be carried out more frequently in Arabic than in Swedish, but they were also quite common in Swedish (Table 5). Storytelling in Swedish was much less frequent though than in Arabic (which is to be expected since we know from Section 4.1 that most parents predominantly spoke Arabic to the child). We could not detect any common tendencies in the backgrounds of the families who carried out joint activities in a particular language very frequently vs. hardly at all. Note however that many parents chose to leave some answers blank; there is a lot of missing information in both languages (see Table 5). In such cases, it may be cautiously assumed that the home activity is never or seldom carried out by parents and child together. Another possible explanation for the large number of missing data could be the fact that the question came at the end of the five-page questionnaire.

One of the language-related home activities we queried was shared book reading, which appeared to be somewhat less common than watching television/videos, singing and storytelling (Table 5). The frequency of shared book reading varied substantially between families. For about half the children, parents reported shared book reading at least once a week (53/100 for Arabic, and 49/100 for Swedish). Twelve families reported that they never did this with their child in Arabic, and 19 that they never did this in Swedish. Moreover, nearly one fifth of the families left this question blank for at least one of the languages.^18^

We were interested to see whether the families who never read books with their child had anything in common, vis-à-vis those who read books together every day. Recall that all parents considered themselves to be fluent in Arabic and all but one parent considered Arabic to be their native language (We did not ask about the parents' literacy levels in Arabic or Swedish though, as this can be a touchy issue.). In families with daily shared book reading in Arabic, most children (12/14) were being read to in Swedish as well, half of them on a daily basis (7/14), and their parents often had a relatively high level of education (tertiary education, 10/14), though some did not. We could not detect any pattern with regard to other factors, such as (parental) length of stay in Sweden. Families who never read books in Arabic with their child often reported that they never read books with them in Swedish either (8/12). Four of the families who never read books in Arabic instead read books in Swedish with their child on a daily basis (these families had often lived in Sweden for 15–30 years). We could not discern any common denominator (such that Sweden-raised parents or parents who had lived in Sweden for a very long time would tend to read to their child more often in Swedish or less often in Arabic). In the families who reported daily shared book reading in Swedish, the parents had a wide range of backgrounds (education, residence length, etc.). However, for those families who never read to their child in Swedish, a few common traits emerged: Disproportionally often, the parents had comparatively low levels of education (primary or lower secondary school), and roughly two thirds of them had relatively short residence lengths in Sweden (0.8–5 years).^19^

In the *longitudinal follow-up* questionnaire and interviews, we also asked about language-related home activities between parent(s) and child. The frequency of these activities varied widely, but the individual families mostly stuck to their earlier activity habits. As in the sample at large, watching television/videos together was again the most commonly reported activity for both Arabic and Swedish, though one family reported that they had stopped doing this in Arabic, and three families now also watched Swedish television and films with the children, which they had not done before, in addition to Arabic. Two such families had also started telling stories in Swedish and listening to and/or singing Swedish songs, which they had not reported 2 years earlier. While at least some shared book reading in Arabic was reported for the majority of children at age 4, by age 6 all 10 children were being read to by their parents in Arabic, and for several this was reported to be done nearly every day. Parent-child shared book reading in Swedish was less common; one family did it on a daily basis, the others less frequently, or never. From the child interviews, it emerged that in one case, an older sibling read Swedish books with the child instead.

Book reading activities at home are dependent on whether the family has access to books. Sweden has many public library branches that not only stock children's books in Swedish but also in Arabic and other minority languages. Borrowing is free of charge. In the interviews with the parents, we explored the issue of book reading and libraries further. Two families mentioned that they had their own children's books at home, mostly Swedish ones. Several families lamented the detrimental effects of the contact and travel restrictions during the COVID pandemic on library visits. That aside, five of the ten families went to the library frequently to borrow children's books, either just Arabic books or books in both Swedish and Arabic. Two families said they never went to the library and two families only rarely did so, for a number of reasons (“because we have books at home,” “because my child isn't into books but prefers to draw,” “because we prefer the mosque”). Whilst the small sample does not allow statistical exploration, it is noteworthy that four of the five frequent library customers were very highly educated, whereas those who never or rarely went to the library mostly had a low level of formal education.

Several parents pointed out that children's books written in Standard Arabic (MSA) could not straightforwardly be read aloud to their child since s/he did not understand MSA but only the family's vernacular, i.e., a regional variety of Arabic such as Lebanese, Palestinian, Syrian. In such cases, the parent often chose to “translate” the book for the child on the fly. For instance, one mother said “We would try to borrow and read both Arabic and Swedish. But my children thought that *fuṣḥa* [MSA] was hard, so I used to translate them to our dialect.” Instead of reading aloud a story book in MSA, parents might improvise and talk about the book with the child in the vernacular, and this was sometimes done with children's books written in Swedish as well. Only one family, frequent library customers, reported that they read books with the child in MSA; these parents were also teaching the child to speak MSA at home. Library visits and shared book reading are conscious efforts to maintain and develop the language(s) of the child.

### 4.5 Enrolment in home language education

Another conscious effort to support home language development is to enroll the child in home language education classes (*modersmålsundervisning* “mother tongue instruction”). In the *large-scale survey*, the parents were asked to specify whether the child attended Arabic home language classes, alone or with others, how often, for how long and who organized them (municipalities, private initiatives or both) (We explicitly asked about Arabic home language instruction, not about religious (Koran/Qur'an) instruction.).

About two-thirds of the children (65/100) attended such classes, whilst 34 did not; attendance however did not distribute evenly across age (see Table 6). A large majority of the 6-and-7-year-olds attended municipal Arabic classes, whereas the 4-and-5-year-olds rarely did; instead a larger share of the 4-and-5-year-olds attended classes run by private organizations or congregations. A likely explanation for this is that following cutbacks of state-funded home language education for preschoolers, municipalities in Sweden nowadays offer these classes only for school-age children (from age 6), which they are legally obliged to. According to the parental questionnaire, most children attended Arabic home language classes once a week, with municipal classes usually lasting for 40 min to 1 h. Privately-run classes typically lasted for 1–4 h. On average, children attended home language education for 1.9 h/week, and most children did so together with other children.

**Table 6:** Arabic home language education, entire sample (100 children), questionnaire responses.

**Age**	**Attendance**	**Municipal**	**Private**	**Both municipal and private**	**None**	**Missing information**
4	46% (10/22)	2	7	1	10	2
5	40% (10/25)	2	7	1	14	1
6	79% (23/29)	18	4	1	6	0
7	92% (22/24)	16	3	3	1	1
Total	65% (65/100)	38	21	6	31	4

We were interested in finding out commonalities between the families whose children were enrolled in Arabic home language education vs. those who were not, but could not discern any tendencies other than older children being enrolled more often than younger ones. Enrolment was no different for children born in Sweden vs. in an Arabic-speaking country, for children of parents raised in Sweden vs. in an Arabic-speaking country, parents' formal education levels, or lengths of residence.

In the *longitudinal follow-up*, in line with the results of the large-scale survey, only three of the children at age 4 were reported to be enrolled in Arabic home language education. By age 6, nine out of ten children attended municipal Arabic classes (45 min to 2 h/week).^20^ One of these children had recently stopped going there because their new teacher spoke a variety of Arabic that the child found hard to understand. Four children attended privately-run Arabic classes in addition to the municipal ones, making their Arabic language instruction sum up to 3–4 h/week. Some of these private language classes, such as Saturday or Sunday school, had been put on hold during the COVID pandemic though. Three children had already started private language classes at age 4; the fourth was not explicitly reported to have private Arabic classes at age 4 but instead frequently attended organized extra-curricular activities with Arabic-speaking peers. This suggests that the parents who had enrolled their children in both private and municipal Arabic classes at age 6 were keen on teaching their children their mother tongue from a very young age.

### 4.6 Enrolment and language practices in (pre)school

In Sweden, preschooling is widespread, extensive and affordable for families irrespective of their socioeconomic background. The children in our sample attended 53 different childcare establishments; Fifty-one went to preschool, mostly for 30–40 h/week (range 15–40 h). The remaining 49 children attended primary school (grade 0 or grade 1). While most children had been to preschool in Sweden, not all of them had done so from an early age because of different histories of migration. In line with Swedish mainstream convention (Statistics Sweden, 2019), most children were enrolled in preschool relatively early. Ninety-one percent of the Sweden-born children (50/55) had started preschool before age 3;0, 64% (35/55) had done so before age 2;0, and many of them as early as 1;0 or 1;3. Children born abroad had generally started preschool later than those born in Sweden. In the entire sample, preschool entry age varied from 1;0 to 5;0 years.

The majority of the preschoolers in our sample (71%, 36/51) went to regular Swedish preschools (see Table 7). Two children attended a bilingual English-Swedish preschool, and 13 were enrolled in preschools with a bilingual Arabic-Swedish profile (25%, 13/51). We could not discern any clear tendency in family background (e.g., formal education, residence length), except that for those who attended Arabic-Swedish preschools, only one child had a Sweden-raised parent and only one spoke Swedish with their parents to any substantial extent. As schooling in Sweden is generally in Swedish, sending one's child to a bilingual Arabic-Swedish preschool is a clear reflection of the parents' wish to maintain and support the development of the home language.

**Table 7:** Enrolment in different types of (pre)schools, entire sample (100 children).

**Age**	**Monolingual Swedish (pre)school**	**Bilingual Arabic/Swedish preschool**	**Swedish school with a Muslim profile**	**Bilingual English/Swedish (pre)school**	**Missing information**
Preschool-age	71% (36/51)	25% (13/51)	–	4% (2/51)	–
School-age	76% (37/49)	–	22% (11/49)	–	2% (1/49)

The 49 school-age children in our sample all attended establishments where schooling officially was in Swedish and followed the Swedish national curriculum (see Table 7). Roughly a fifth of them, 22% (11/49), were enrolled in independent schools with a Muslim profile and many staff members spoke Arabic there. Sending one's child to such a school might be a deliberate act of parental language planning. Families whose children attended such a school generally had some things in common: None of the children spoke predominantly Swedish to their parents. Most parents had lived in Sweden for only a short time (1–4 years) and had relatively low levels of formal education (primary or lower secondary school), or left the questions on education blank (The reverse did not hold though, many other parents with short residence lengths and/or low levels of formal education sent their children to regular municipal schools without a religious profile.). A couple of parents with longer residence lengths and higher education who sent their child to Muslim-profile schools taught at these institutions themselves.

Many (pre)schools were located in multilingual urban neighborhoods, and sometimes Arabic was a prominent language there. We noticed during our school visits that often a large proportion of the intake appeared to be children with a history of migration and with varying levels of proficiency in Swedish. While the official language of communication in the (pre)schools was Swedish, the extent to which monolingual Swedish or multilingual staff were employed seemed to vary considerably. Preschool staff were not always proficient in Swedish. We also observed that some staff members spoke languages other than Swedish on the premises during school hours, in conversation with colleagues, parents or children. Taken together, these observations suggest that multilingual practices in the children's (pre)schools are commonplace. Our survey and interviews with the parents confirmed these impressions. In the questionnaires, we explicitly asked whether the child had a (pre)school teacher or other member of staff who spoke Arabic with them. In the *large sample*, parents reported this to be the case for a quarter of the children (24/100), and thus not only for the 13 children who were schooled bilingually in Arabic and Swedish.

In the *longitudinal follow-up*, due to the COVID pandemic restrictions, it was not possible to conduct school observations as originally intended, but the family interviews informed us about the children's language input at (pre)school. According to the parents, most children (8/10 at age 4; 7/10 at age 6) regularly interacted with at least one Arabic-speaking staff member. The majority of the children (7/10) also mentioned that they had at least one Arabic-speaking peer in class. At age 4, three children had been enrolled in bilingual Arabic/Swedish preschools. By age 6, one child attended a bilingual English/Swedish school and nine children attended monolingual schools where Swedish was the language of instruction. However, three families mentioned that there were several Arabic-speaking staff members at their children's current school (a school with the aforementioned Muslim profile).

In sum, for our sample, the use of Arabic appears to be tolerated and/or welcomed on many (pre)school premises. Whilst some families sent their child to a (pre)school with a particular profile that welcomed Arabic, other children attended the local mainstream (pre)school. There too, Arabic may be spoken because intake often includes Arabic-speaking children and may also include Arabic-speaking staff, which may be conducive to home language maintenance.

### 4.7 Parental language attitudes and beliefs

As previously mentioned, a high proportion of children in the sample attended Arabic home language education classes, especially at age 6–7. Moreover, a number of parents enrolled their child in a bilingual Arabic-Swedish preschool or a school with Arabic-speaking teachers. Both of these findings point to deliberate language management and positive parental attitudes toward the child learning Arabic.

In the *large sample*, the parents were asked which language they considered to be the most important for their child to become proficient in. In most cases (81/100), the parents answered that Swedish and Arabic were equally important. Such an answer is expected and it is also the “politically correct” answer, as it aligns with Sweden's official language policy, which states that children should develop both their home language and Swedish. However, a sizeable number of parents answered differently: 12/100 considered learning Arabic to be more important for their child than Swedish, 2/100 regarded learning Swedish as more important than Arabic, and 4/100 stressed the importance of learning English in addition to Arabic and Swedish. For one child, no such information was available.

We were interested to see whether these parental language attitudes translated into action. Almost all parents reported that they communicated with their child predominantly in Arabic (Section 4.1), which suggests that they were keen to provide the input their child needs to develop their home language. Regarding Arabic home language education, only 58% (7/12) of the families who considered proficiency in Arabic to be more important than in Swedish sent their child to Arabic lessons. It is noteworthy that all but one of these children were so young (age 4–5) that municipal home language education was not available for them, so these families had opted for privately-run Arabic home language classes. Six of the 12 families who considered Arabic to be more important than Swedish had moreover chosen to enroll their child in a bilingual Arabic-Swedish preschool. Unsurprisingly, the two families who considered Swedish to be more important than Arabic had not enrolled their child in any Arabic classes. At least to some extent then, the parents' language ideology seems to match their language practices (although the numbers are too small for statistical analysis).

In the questionnaire, we did not query *why* the parents believed that it was important for their child to learn a certain language. When interviewing the parents 2 years later in the *follow-up*, we did ask this question. All parents expressed a strong wish for their child to become proficient in Arabic. (“Yes!”, “Yes absolutely!”, “Yes of course!” were their responses throughout.) The prime motivation given was being able to stay in touch and communicate with their immediate and extended family circle, as many relatives lived outside of Sweden. Only one parent thought that communication could be achieved in other ways too: “Yes, I want him to connect with family in [country of origin]. Although, more and more of the younger ones are currently switching to English when speaking to him. So children and adults are able to communicate anyway regardless if my child is very fluent in Arabic or not.” Another motivational factor mentioned was ethnocultural and religious affiliation. Some parents were keen on their child to be able to read religious texts and participate in liturgical services in Arabic in the future. One parent also mentioned that proficiency in Arabic was needed if the family were to return to their country of origin (“we might not stay in Sweden forever”).

In the large-scale survey, parents were asked whether they had ever felt any anxiety about their child's language development and why, and whether they had sought professional help or advice. Most families (84/100) reported no such anxiety, two left this question blank, while 14 parents reported that they had felt anxious in the past or did so now. In some instances, this was because of the child's late onset of speech or the parents' perception that the child's language development was slow (such as not being able to combine words at age two, or not being able to pronounce certain speech sounds at age 4). Most of these problems had resolved since then. Eight families had been anxious because of the child growing up bilingual. The responses to the open-ended question showed that they were concerned about the following: How would the child cope at school in Sweden when the family did not speak Swedish well, or when the child mainly communicated in Arabic with playmates and relatives? Would the child catch up with Swedish-speaking age peers even though s/he had started preschool late? Alternatively, the parents worried about their child mixing the two languages, or about the child's Arabic being full of mistakes and not developing any further. Only two families had consulted a speech-language therapist (SLT) about these issues. Several other families reported that they had been referred by child healthcare nurses to a speech-language therapist. According to the parents, the SLT had reassured them that the child's language development was normal.

In the *longitudinal study*, none of the 10 families expressed any anxiety about their child's language development at age 4. Two years later, when asked directly in the interview, two families indicated that they were anxious about their child's development in Swedish, and another family was concerned that their child refused to speak to the parents in Arabic. We also asked whether the parents were happy and content with their child's language use in home and with their proficiency in Arabic, and most of them (7/10) were. Three parents expressed discontent because the child no longer spoke Arabic to the parents and only replied in Swedish or because the child did speak Arabic but not in a “proper” way.

In the interviews, we also asked whether the parents had ever actively sought advice on bringing up their child bilingually. Interestingly, nine out of ten families said they had not asked for advice but that it was proffered to them nonetheless. Half of them had been recommended to always speak their native language (Arabic) at home in order to maximize input in the home language; such advice was proffered by other Arabic-speaking parents in Sweden or by the school. The only family who reported to have actively sought advice about bringing up their child bilingually had done so because the child's Swedish was weak at the time and the child was having trouble coping at preschool. The parents consulted the child's preschool teacher who counseled them to speak more Swedish with the child. None of the other families in the longitudinal sample reported having received similar advice. Some parents felt that bringing up one's child bilingually was not a matter to be advised on by outsiders, but that the matter was being discussed between spouses (“We discuss how important it is for us to speak Arabic at home, since we know that the child will learn Swedish in the society,” “We don't speak about this topic with other parents or teachers. But we have a lot of friends who speak Arabic with their kids”).

When asked which advice they would give to other Arabic-speaking parents in Sweden, all of them said that parents should speak Arabic with the child and maximize exposure to Arabic in the home (“Speak the mother tongue at home!”, “I tell them not to forget their mother tongue!”). Often no explicit rationale for this advice was given, though in some cases it was pointed out that the child needed Arabic to be able to communicate with extended family. Two parents mentioned that although they believed that speaking Arabic to the child in the home was the right thing to do, they knew of other families who did not. As one mother said, “I would tell them to speak Arabic at home, and leave off Swedish at home. But some parents are not convinced and prefer to speak Swedish so that the children do not become weak as them.” One parent believed that parents should strengthen the child's mother tongue, “since then it is easier to build upon it new languages.”

Many of the parents were of the opinion that society would eventually teach their child Swedish, whereas it was their job as parents to teach the child Arabic (e.g., “we advise each other to continue speaking Arabic, since the children will eventually learn Swedish”). However, two families stressed that parents should also pay close attention to their children developing good Swedish skills (“Swedish is just as important as Arabic here”). As one mother pointed out: “Also Swedish is very important, in order to communicate. The language is important to feel that you belong. I would advise anyone to learn the local language with their children, but also to keep the mother tongue.”

## 5 Discussion

This study has used parental questionnaire and interview data to investigate the language practices and beliefs of the families of 100 Arabic/Swedish-speaking children in Sweden. Whilst families were diverse regarding their history of migration, formal education, country of origin and length of residence in Sweden, parents valued the home language Arabic highly and wanted to pass it on to their children. This maintenance orientation is perhaps not very surprising and may feel completely natural to parents when they have immigrated to Sweden relatively recently and/or are not fluent in Swedish. However, when the parents are bilinguals themselves, having grown up in Sweden and/or having lived in Sweden for a long time, the choice of transmitting the heritage language to the younger generation is generally deliberately made. In the eyes of the majority society though, the decision to maintain a language that is not the majority language is not necessarily perceived as a neutral act, but can be perceived as a “political” one (Abdelsayed and Bellinzona, 2024).

In line with research on a different immigrant minority-language group in Sweden, namely Turkish-heritage families (Aktürk-Drake, 2017, 2018), and in line with research on Arabic-heritage families in other settings (e.g., Bentahila, 2011; Eid, 2019; Yousef and Taylor-Leech, 2018; Attaallah, 2020; Alraddadi, 2021; Abdelsayed and Bellinzona, 2024), the findings of the present study suggest that Arabic-speaking families in Sweden ascribe a high symbolic value to their heritage language, and that they have strong language maintenance ideologies as well as strong bilingualism ideologies.

Nearly all parents in the present study (98%) considered it important that their child becomes proficient in Arabic, alongside the majority language Swedish. Parents indicated that they viewed Arabic/Swedish bilingualism as an asset, but also that they considered it their job to transmit and support the development of the minority language Arabic, while school and mainstream society were seen as responsible for teaching the child the majority language Swedish. This explicitly expressed opinion fits well with the reported language practices: In nearly all families, both parents spoke only or mostly Arabic to each other (90%) and to the child (79%). The majority of children (68%) were reported to speak mostly Arabic to their parents as well, though not quite as frequently as parents spoke Arabic to them. The pattern observed in the cross-sectional data also held for the smaller longitudinal study, where 10 of the 4-year-old children were followed up 2 years later, at age 6. By then, some children had moved toward speaking more Swedish with their parents (and with their siblings), although the parents strongly preferred them and tried to make them speak Arabic. However, these language management efforts sometimes clashed with child agency.

Classic assimilationist approaches have suggested that minority-language maintenance and shift can be captured by generational models (Fishman, 1972; Veltman, 1988; Rumbaut, 2004). Thus, the children of Arabic-speaking parents who came to Sweden as adults (Generation 1) should develop stronger Arabic than the children of parents who themselves grew up in Sweden (Generation 2). As the children in the present study are only 4–7 years old and still developing their language skills, it is impossible to test such predictions with the current data.^21^ However, we did find that only 2 of the 12 children in our sample who had a Sweden-raised parent were reported to speak a lot of Swedish to their parents, whilst 29 children without any Sweden-raised parent were also reported to speak a lot of Swedish to their parents. This could suggest that a purely generational approach to minority language use in the home is too simplistic (Fishman, 1991; Pearson, 2007) and that other factors influence children's language use with their parents in the home, in line with sociolinguistic approaches that focus on child agency and family language policy (Lanza, 1997; Luykx, 2005; Schwartz, 2010).

In the present study, in both the cross-sectional and the longitudinal sample, language use between siblings was somewhat different from parent-child interactions, as only 39% were reported to communicate mostly in Arabic with their siblings, and the majority used both Arabic and Swedish, or mostly Swedish, a shift over time that also became evident through the longitudinal follow-up. This shift toward speaking more Swedish with siblings echoes several studies from other heritage-speaker populations that show that children tend to speak the majority language more often with their siblings than with their parents (e.g., de Houwer, 2007; Barron-Hauwaert, 2011; Bridges and Hoff, 2014; Aktürk-Drake, 2017; Sorenson Duncan and Paradis, 2020; Abdelsayed and Bellinzona, 2024). A similar shift toward using more Swedish with siblings has also been found for 4-to-7-year-olds growing up in Sweden with Turkish as their heritage language. They were investigated with the same methodology as the present study, in a parallel subproject of the larger child multilingualism project that the present study is a part of (BiLI-TAS, Bohnacker, 2022, 2023).

From a methodological viewpoint, we could note that the interviews provided insights that complemented the information we culled from the questionnaires in important ways and occasionally were at odds with it. In particular, this concerned the child's use of Swedish at home. From the interviews, it emerged that for several children, the child's use of Swedish at home was more extensive or had shifted toward more Swedish than the written questionnaire responses suggested (recall Table 4). In conversation with the interviewer (the second author), a native speaker of Arabic whose background partially overlaps with that of our participants (as she herself immigrated to Sweden during childhood and now also raises a bilingual family), the mothers recounted aspects of child agency that the quantitative questionnaire responses did not show: In some families, the child put up resistance, challenged or ignored aspects of the parents' desired family language policy, namely that of Arabic being spoken in the home, and insisted on speaking Swedish to the parent and/or with the siblings, within or out of earshot (reminiscent of what has been described elsewhere in the literature, e.g., Luykx, 2005; Smith-Christmas, 2020). The parents handled such situations in different ways, by “forcing” the child to speak Arabic, letting it pass, or “giving in” and switching to Swedish themselves (cf. Lanza, 1997, 2007; Gafaranga, 2010). It would be interesting to investigate these aspects further with more observational data on everyday interactions, as our findings are based on reported data only.

The interviews also complemented the quantitative results from the questionnaires in other important ways. For instance, it only emerged during the interviews that the parents and children were in regular contact with grandparents and other relatives, via phonecalls, videocalls and visits. Wanting to be able to communicate with loved ones who do not speak the majority language can be a powerful motivation for continued minority-language use and maintenance (Et-Bozkurt and Yagmur, 2022). Children and adolescents tend to make an effort to use the home language with their grandparents, because of their limited knowledge of the majority language and/or because of their respected status in the family (Pauwels, 2016: p. 125; Et-Bozkurt and Yagmur, 2022; Abdelsayed and Bellinzona, 2024). In the present study, several parents said that staying in contact with relatives was their main reason for pushing the child to learn Arabic, and thanks to recent advances in telecommunication, grandparents and other relatives could be regularly co-present in the lives of the families.

Another case in point was (shared) book reading and joint visits to the library. Such activities with the child can nurture socioemotional bonds, build language and literacy skills and thus aid minority-language maintenance. Indeed, “with literature and popular culture the value of a language can be enhanced so that the child will seek more input through that medium” (Pearson, 2007: p. 403). The interviews yielded data on literacy practices that remained hidden in the questionnaire data, such as the parents translating children's books written in MSA into the family vernacular (e.g., Lebanese, Syrian) when reading with the child. Triangulating family background information and interview data, we also found that parents who frequently went to the library with their child tended to be very highly educated, whereas those who never or rarely went to the library mostly had a low level of formal education. A similar pattern in library customs regarding children's books has been described for Turkish-speaking families in Sweden (Bohnacker, 2022). However, caution is advised because of small sample size.

Most families in our sample sent their child to preschool from an early age, in line with Swedish mainstream convention (Statistics Sweden, 2019). One fifth enrolled their child in an independent bilingual Arabic-Swedish preschool, or in an independent primary school with a Muslim profile where many staff spoke Arabic, i.e., sociolinguistic environments that support the home language (cf. Schwartz, 2010). Sending one's child to such an establishment might be a conscious act of parental language planning, as the default option would be for the child to attend the local municipal monolingual Swedish (pre)school. However, the bilingual preschools and primary schools with a Muslim profile were located in areas where many Arabic speakers lived. Especially when parents are newcomers with little knowledge of Swedish, it may be convenient to send their child there, give a sense of relief that they can communicate with teachers and school management in their native language, and assure them that their child will not be left out because they cannot communicate their needs to the school (Recall from Section 4.6 that most of the families who sent their children there had not been in Sweden for very long.).

Irrespective of the profile of the school, we noticed that Arabic was spoken, at least to some extent, on many of the premises of the 53 (pre)schools we visited. This could be due to where we gathered our sample. When there are many pupils who speak the same minority language, (pre)schools tend to hire staff who also speak that language to ease communication, which could be interpreted as a sign that minority languages are welcomed and valued. Bear in mind though that our participants largely came from multilingual, multicultural urban areas. In other areas, the language setting and language policy of the (pre)school may be different.

This brings us to the issue of sampling. As mentioned in the Methods section, random sampling from the national population register was not feasible, as no statistics are kept on whether a resident of Sweden speaks Arabic or not. Whilst the questionnaire survey does cover the families of 100 children, we only recruited from urban settings in eastern central Sweden. This was done in part for logistic reasons, but also because this is a region where a large share of the Arabic-speaking population of Sweden lives (Bohnacker, 2017). We cannot be sure how representative our participants' language practices, experiences and opinions are of Arabic-heritage families in the entire country. Children growing up in less heavily multicultural urban areas (or in rural ones) may attend (pre)schools with a lower multilingual and/or lower Arabic-speaking pupil intake, and there might be fewer, if any, Arabic-speaking staff on hand.

Also, since the broader research project (BiLI-TAS) was geared to investigating children's expressive language skills in both languages, we only included families whose child could speak at least some Arabic and at least some Swedish. This means that the survey does not cover the language practices and beliefs of Arabic-heritage parents whose children do not speak any Arabic, who may have other views than the participants of the present study.

Moreover, because of travel and contact restrictions during the COVID pandemic, a longitudinal follow-up could only be carried out with a small number of families (10 out of the 22 families whose children were 4 years old at the time of the first data collection). This limits the generalisability of the findings from the follow-up study.

The present work should be extended by investigating family language practices over a longer stretch of time. A second follow-up study is currently under way, which will shed light on (possibly) new family dynamics, as the children get older and are schooled further in the Swedish system, as they are increasingly exposed to English via school and the media, and as new siblings are born.

As mentioned above, concerning family language practices we did not have access to what parents and children actually do, only to what they say they do. Therefore, the present study could be profitably complemented by careful observations of actual day-to-day interactions between family members (Lanza, 2007; King et al., 2008; Gafaranga, 2010), which should include dyadic parent-child conversations but also multi-party family interactions.

In the interviews, parents reported that their efforts to maintain the home language were not only tolerated but often explicitly welcomed and endorsed by educators in mainstream institutions such as preschools, schools and child health services. For instance, teachers, schools and speech-language therapists were more or less unanimously reported to advise parents to maximize Arabic input to their child in the home (except in one case). The rationale behind such advice was that a well-developed minority language would help the child to learn other languages, including the majority language Swedish, more easily. This is a popularized version of the interdependence hypothesis, according to which proficiency in the first language strengthens and speeds up the acquisition of the second language (e.g., Skutnabb-Kangas and Toukomaa, 1976; Cummins, 1979, 1991; Thomas and Collier, 1997). It remains to be seen whether a larger-scale investigation of the advice that minority-language parents receive from educators concerning their children's bilingualism would yield the same result. For instance, would parents of minority-language children who attend a school with a largely monolingual Swedish intake be given different advice, such as trying to maximize exposure to Swedish at home? Or would the type of advice given be influenced by the teacher's training and background (Mary and Young, 2020)? Interestingly, in a study of home-language maintenance in a different ethnolinguistic group in Sweden, (Bohnacker 2023) found that Turkish-heritage parents were given the same kind of advice by teachers and other Swedish educational experts as the parents interviewed in the current study, i.e., to maximize Turkish input to their child in the home. This points to a favorable orientation of the host society toward minority-language maintenance. When parents and the overall language community consider heritage languages as a core value, language maintenance becomes much more sustainable (Et-Bozkurt and Yagmur, 2022).

## Data Availability

The datasets presented in this article are not readily available because the present study is part of a larger, ongoing research project. For ethical reasons, the original raw dataset cannot be shared in full as this would violate the protection of human subjects. For data supporting the reported results, please consult the corresponding author. Requests to access the datasets should be directed to Ute Bohnacker, ute.bohnacker@lingfil.uu.se.

## Footnotes

1 Arabic-speaking countries are considered to be countries where Arabic is the official language and the primary language of instruction in public schools: Algeria, Bahrain, Egypt, Iraq, Jordan, Libya, Kuwait, Lebanon, Mauretania, Morocco, Oman, Palestine, Qatar, Saudi-Arabia, Sudan, Syria, Tunisia, United Arab Emirates and Yemen.

2 Regional varieties that originate from distant enough geographical areas can be mutually unintelligible. For instance, a speaker of Levantine Arabic (an umbrella term for varieties spoken in the Middle East) and a speaker of a Maghrebi (North African) variety may only partly understand each other, unless they modify their speech to resemble the standard variety as a common code.

3 By *language maintenance* we mean “the continuing use of a language in the face of competition from a regionally and socially more powerful language” (Mesthrie and Leap, 2009: p. 245). By *language shift* we mean that one language is replaced by another language as the “primary means of communication and socialization” (ibid.).

4 The *majority* or *societal language* is the language spoken by a socially and/or economically dominant group in a national context. *Home languages* are defined as languages that are not the majority language of society and are spoken by the child's family and/or close community. The terms home language, minority language and heritage language are used interchangeably here.

5 The term *family language policy* was first used by (Luykx 2003, 2005) and popularized by (King et al. 2008).

6 *Attitudes* and *beliefs* are related concepts and the terms are often used interchangeably. There exists a wealth of definitions for these terms. In social psychology, beliefs refer to convictions people hold about the world around them, whilst attitudes refer to evaluations or feelings towards a particular object/person/idea/event (American Psychological Association, 2018). Attitudes can also be seen as expressions of a particular belief or set of beliefs (Eagly and Chaiken, 1993). In the present study, the terms are used interchangeably.

7 On the contrary, researchers have argued that when teachers encourage parents to speak with their child in a language they are not proficient in, this may force parents to interact at a level far below their actual level of cognitive maturity (Cummins, 1981, 1991; Thomas and Collier, 1997).

8 For instance, the far-right “Sweden Democrats” (*Sverigedemokraterna*) campaigned at national level with the slogan *Vi ska inte lägga en krona på att lära barn arabiska* (“we won't pay one penny for teaching children Arabic”) on their official accounts on social media and on billboards across the country in 2023.

9 There is also a (tangential) festschrift paper (Wardini, 2017) that reports on a survey administered to 25 second-generation (Sweden-born) speakers of Arabic whose parents were born in an Arabic-speaking country. The respondents were 16–18 years old and attended municipal Arabic classes at two schools in Stockholm. The questionnaire tackled language attitudes and identity, but other than Arabic contributing positively to identity, no clear patterns emerged.

10 The families of 117 children had originally signed up to participate, though eventually only 100 could be included, due to dropouts along the way or when it became clear that the child had not yet reached their fourth birthday, had very rudimentary knowledge of Swedish or Arabic, showed only minimal signs of comprehension, or could not do any of the language tasks.

11 A reviewer asks which parent filled in the questionnaire, the mother or the father. This varied. We did not ask about the mother or the father directly, but about parent/guardian 1 and 2, as is common in Sweden. Sometimes parent 1, and sometimes parent 2 filled in the questionnaire, and sometimes the parents did it together.

12 With hindsight, we could have tried harder to interview more parents remotely; however, since the main objective of the BiLI-TAS project was to collect follow-up language data from the children (and not only to interview the parents), we focused on re-recruiting the families of children that we could meet in person.

13 Five years later, a second longitudinal follow-up was carried out with the families of 23 children (when they were 9–10 years old). The results of this second follow-up are not reported here as data analysis is still ongoing.

14 Here and in what follows, by “Arabic” we mean colloquial (regional) varieties of Arabic.

15 One child spoke mostly Swedish to one parent and equal amounts of Arabic and Swedish to the other; 13 children spoke both Arabic and Swedish evenly with both parents; 7 children spoke speak mostly Arabic to one parent and equal amounts of Arabic and Swedish to the other parent. Only in one household was the child reported to speak mostly Arabic to one parent and mostly Swedish to the other (this was the abovementioned parent in the sample who preferred to speak Swedish with their spouse and child).

16 The parents of four children (BiAra5-06, BiAra6-17, BiAra7-17, BiAra7-23) reported that their child also occasionally spoke English with their sibling(s) in addition to either mostly Arabic (*N* = 2), mostly Swedish (*N* = 1), or both languages (*N* = 1). Since in none of the four cases English was reported as the only or predominant language spoken between the siblings, these cases were not coded as a separate “other” category.

17 The parents were asked whether their child heard Arabic from extended family, friends and (pre)school staff in the form of a multiple-choice question where they could tick one or several input givers. Not having selected an answer thus need not indicate that the child does not hear Arabic from this type of input giver, but could also be due to carelessness while filling in the questionnaire.

18 When filled in for one language but left blank for the other, this may indicate that the parents do not read books with the child in the language left blank.

19 Recall that the average length for parental residence was 9.7 years, with a range of 0.8–40 years.

20 The tenth child was reported to be taught Standard Arabic (MSA) by the parents at home, both at age 4 and 6.

21 Also, a larger sample would be needed that includes families from Generation 1–3, which may be difficult to achieve as immigration from Arabic-speaking countries to Sweden is relatively recent.
