# Supplementary material for: Arabic as a home language in Sweden: family language practices and beliefs
Source: Front Psychol. 2025 Dec 16;16:1719805. doi: 10.3389/fpsyg.2025.1719805 (PMC12747834; doi:10.3389/fpsyg.2025.1719805)
Supplement: Supplementary file 1 [file Data_Sheet_1.PDF]

BiLi TAS [Bilingualism &Language Impairment | Turkish Arabic Swedish]

UPPSALA UNIVERSITET

استمارة اسئلة إلى حامل حق حضانة الطفل:
دراسة حول مقدرة الطفل على السرد (رواية القصص)

1. **اسم الطفل/ الطفلة:** ______________________

2. **تاريخ الميلاد:** ______________________

3. **الجنس** [ ] فتاة [ ] صبي

4. **هل هذا طفلك الاول؟ الثاني؟ الثالث؟ ضع علامة × على الرقم الصحيح.**
[ ] 1 [ ] 2 [ ] 3 [ ] رقم آخر ______

5. **ما هي اللغات التي يتكلمها طفلك الان؟**
[ ] السويدية [ ] العربية [ ] لغة اخرى، ما هي؟ ______________

[ ] العراقية [ ] اللبنانية [ ] لهجة اخرة
[ ] السورية [ ] الفلسطينية ______________

6. **في اي دولة ولد طفلك؟**
[ ] في السويد [ ] في دولة تتحدث اللغة العربية، ما هي؟ ______________ [ ] في دولة اخرى، ما هي؟ ______________

7. **منذ متى يسكن طفلك في السويد؟**
[ ] منذ الولادة [ ] وقت آخر: عدد السنوات __________

8.أ. **هل يذهب طفلك إلى المدرسة؟**
[ ] نعم [ ] كلا

إذا نعم

اسم المدرسة: ______________________

عمر طفلك عند الدخول في المدرسة: سنة _____ شهر _____

اي نوع من المدارس؟
[ ] احادية اللغة سويدية
[ ] ثنائية اللغة سويدية / عربية
[ ] أخرى __________

هل ذهب طفلك إلى المدرسة التمهيدية (الحضانة) "dagis"؟
[ ] نعم [ ] كلا

نوع المدرسة التمهيدية (الحضانة)
[ ] احادية اللغة سويدية
[ ] ثنائية اللغة سويدية / عربية
[ ] أخرى __________

عمر طفلك عند الدخول في المدرسة التمهيدية
السنة __________ الشهر __________

فترة البقاء (الدوام) بالأسبوع: __________ ساعة

هل كان هنالك انقطاع طويل (على سبيل المثال بسبب ولادة طفل اخر)؟
[ ] كلا [ ] نعم ________ شهر

ما هي اللغة التي يتحدث بها الموظفون مع طفلك؟
____

إذا كلا

هل يذهب طفلك إلى مدرسة تمهيدية؟
[ ] نعم [ ] كلا

نوع المدرسة التمهيدية
[ ] احادية اللغة سويدية
[ ] ثنائية اللغة سويدية / عربية
[ ] أخرى __________

عمر طفلك عند الدخول في المدرسة التمهيدية
السنة __________ الشهر __________

فترة البقاء (الدوام) بالأسبوع: __________ ساعة

هل كان هنالك انقطاع طويل (على سبيل المثال بسبب ولادة طفل اخر)؟
[ ] كلا [ ] نعم ________ شهر

ما هي اللغة التي يتحدث بها الموظفون مع طفلك؟
____

اسم المدرسة التمهيدية:
____

8.ب. هل يذهب طفلك الى دار أوقات الفراغ "fritids" ؟

[ ] نعم [ ] كلا

9. ما رأيك في التطور اللغوي عند طفلك؟

العربية [ ] مبكر [ ] طبيعي [ ] متأخر

السويدية [ ] مبكر [ ] طبيعي [ ] متأخر

10. كم كان عمر طفلك عندما قال كلمته الاولى؟

بالعربية: سنة ______ شهر ______ **بالسويدية**: سنة ______ شهر ______

11. كم كان عمر طفلك عندما قال عدة كلمات متتابعة؟

بالعربية: سنة ______ شهر ______ **بالسويدية**: سنة ______ شهر ______

12. هل كنت في مرة من المرات قلقاً على تطور طفلك اللغوي؟

[ ] كلا [ ] نعم، متى ولماذا؟ ____

13. هل يوجد احد في العائلة لديه صعوبات لغوية و/او صعوبات بالقراءة والكتابة؟

[ ] كلا [ ] نعم، بأي طريقة؟ ____

14. هل تعتقد أن تطور طفلك اللغوي يختلف عن اخوانه واخوته او اقربائه او اصدقاء اللعب؟

[ ] كلا [ ] نعم، بأي طريقة؟ ____

15. هل كان لطفلك في مرة ما اتصال مع اخصائي نطق؟

[ ] كلا [ ] نعم، لماذا؟ ____

16. هل كان لدى طفلك مشكلة بالسمع؟

أ. ضعف بالسمع

[ ] كلا
[ ] نعم

ب. التهاب متكرر بالأذن

[ ] كلا
[ ] نعم، كم مرة؟ ______________
[ ] طفلي لديه انبوب

17. هل يسمع طلفك بشكل طبيعي الان؟

[ ] نعم [ ] كلا

18. **معلومات حول الوالدين / حامل حق حضانة الطفل**

**المعرفة اللغوية**	هل **العربية** هي لغتك الام؟ **إذا كلا**، بأي مستوى تتحدث اللغة؟	هل **السويدية** هي لغتك الام؟ **إذا كلا**، بأي مستوى تتحدث اللغة؟	ما هي اللغات الاخرى التي تجيدها؟
حامل حق حضانة الطفل/ الوالد 1			
حامل حق حضانة الطفل/ الوالد 2			

**الخلفية**	اين ولدت؟ (الدولة والاقليم)	اين كبرت وترعرعت؟ (الدولة والاقليم)	كم المدة التي سكنتها في السويد؟
حامل حق حضانة الطفل/ الوالد 1			
حامل حق حضانة الطفل/ الوالد 2			

**التعليم/ المهنة**	التعليم	في اي دولة اكملت الجزء الاكبر من تعليمك؟	المهنة
حامل حق حضانة الطفل/ الوالد 1			
حامل حق حضانة الطفل/ الوالد 2			

19. **ما هي اللغة التي تتحدث <u>انت</u> بها مع طفلك؟ (ضع علامة × في المكان المناسب)**

	تقريباً فقط العربية	العربية غالباً، احيانا السويدية	العربية 50%، السويدية 50%	السويدية غالباً، احيانا العربية	تقريبا فقط السويدية	شيء اخر:
حامل حق حضانة الطفل/ الوالد 1						
حامل حق حضانة الطفل/ الوالد 2						

20. **ما هي اللغة التي تتحدثون بها أنتم الوالدين/ حامل حق حضانة الطفل مع <u>بعضكم البعض</u>؟**

____

21. **ما هي اللغة التي يتحدث بها <u>طفلك</u> معك؟ (ضع علامة × في المكان المناسب)**

	تقريبا فقط العربية	العربية غالباً، وأحياناً السويدية	العربية 50%، السويدية 50%	السويدية غالباً، وأحياناً العربية	تقريبا فقط السويدية	شيء اخر:
حامل حق حضانة الطفل/ الوالد 1						
حامل حق حضانة الطفل/ الوالد 2						

22. ما هي اللغة التي يتحدث بها طفلك مع اخوانه واخواته؟

[ ] ليس لطفلي اخوان واخوات
[ ] العربية غالباً
[ ] السويدية والعربية
[ ] السويدية غالباً
[ ] لغة أخرى ____________

23. من اي عمر بدأ طفلك يسمع السويدية بشكل منتظم؟ (ضع علامة × في المقياس)

الولادة	1 سنة	2 سنة	3 سنة	4 سنة	5 سنة	6 سنة	7 سنة

24. من اي عمر بدأ طفلك يسمع العربية بشكل منتظم؟ (ضع علامة × في المقياس)

الولادة	1 سنة	2 سنة	3 سنة	4 سنة	5 سنة	6 سنة	7 سنة

25. أ. هل يسمع طفلك العربية من ... ؟

[ ] الاخوة والاخوات
[ ] الاقارب / اصدقاء العائلة
[ ] اصدقائه
[ ] الكتب
[ ] التلفاز / الافلام / الحاسوب / الهاتف / الموسيقى
[ ] اخر

25. ب. هل يسمع طفلك لهجة عربية غير تلك التي تتكلمونها في المنزل؟

[ ] كلا
[ ] نعم. أية لهجة وأين يسمعها؟ ____

____

25. ت. هل يسمع العربية الفصحى في حياته اليومية؟ (مثلاً: برامج الأطفال على التلفاز)

[ ] كلا
[ ] نعم. أين يسمعها؟ ____

____

26. كم يسمع طفلك كِلتا اللغتين في حياته اليومية؟ (ضع علامة × في المقياس)

السويدية 5% العربية 95%	السويدية 20% العربية 80%	السويدية 40% العربية 60%	السويدية 50% العربية 50%	السويدية 60% العربية 40%	السويدية 80% العربية 20%	السويدية 95% العربية 5%

[ ] آخر ____________________

27. ما هي أفضل لغة يتحدث بها طفلك الان؟

[ ] العربية
[ ] السويدية
[ ] الاثنين بنفس الجودة
[ ] لغة أخرى ____________________

28. هل يفضّل طفلك ان يحكي إحدى اللغتين على الاخرى؟

[ ] كلا
[ ] نعم، ما هي؟ ____________________

29. قيّم معرفة طفلك اللغوية من خلال أن تشير اختيار واحد.

	جيد جداً	جيد	نوعا ما جيد	سيء	سيء جداً
مدى استيعاب طفلك **العربية؟**					
مدى استيعاب طفلك **السويدية؟**					
مدى تحدث طفلك **للعربية؟**					
مدى تحدث طفلك **للسويدية؟**					

30. ما هو الاهم بالنسبة لك أن يتعلمه طفلك؟

[ ] السويدية هي الاهم [ ] الاثنتين مهمتين
[ ] العربية هي الاهم [ ] أخرى: ______________________

31. هل يحصل طفلك على نوع من تدريس أو دعم اللغة الام في العربية؟

[ ] نعم، معلم اللغة الام التابع للبلدية [ ] كلا
[ ] نعم، بشكل خاص (مثلا: عبر جمعيات او مجموعات الدينية)

إذا نعم، اذكر عدد الساعات في الاسبوع ______________
إذا نعم، هل هو مع اطفال اخرين [ ] نعم [ ] كلا

32. هل يخصص طفلك وقت لنشاطات اوقات الفراغ المنظمة (مثل، الرياضة والموسيقى)

[ ] نعم [ ] كلا
إذا نعم، بأي لغة تُمارس هذه النشاطات؟

	كل يوم	على الاقل مرة في الاسبوع	نادراً
العربية			
السويدية			
لغة اخرى، اذكرها ____________			

33. كم مرة قمت بهذه النشاطات التالية مع طفلك خلال الشهر الماضي؟

	العربية				السويدية			
	ابداً	مرتين بالشهر	مرة أو مرتين بالأسبوع	كل يوم تقريباً	ابداً	مرتين بالشهر	مرة أو مرتين بالأسبوع	كل يوم تقريباً
**سرد القصص**								
**القراءة**								
**الاستماع إلى الاغاني**								
**مشاهدة التلفاز /فلم/الكومبيوتر**								

34. **هل طفلك معتاد أن يروي لك حكايات وقصص؟**

[ ] نعم، غالباً
[ ] نعم، احياناً
[ ] نعم، ولكن نادراً
[ ] كلا

35. **هل يستطيع طفلك القراءة والكتابة:**

[ ] كلا
[ ] نعم، يقرأ ويكتب اسمه
[ ] نعم، كلمات/جمل بسيطة بالعربية
[ ] نعم، بطلاقة بالعربية
[ ] نعم، كلمات/جمل بسيطة بالسويدية
[ ] نعم، بطلاقة بالسويدية

36. **هل هنالك شيء تريد أن تضيفه؟**

شكراً
