# Supplementary material for: Arabic as a home language in Sweden: family language practices and beliefs
Source: Front Psychol. 2025 Dec 16;16:1719805. doi: 10.3389/fpsyg.2025.1719805 (PMC12747834; doi:10.3389/fpsyg.2025.1719805)
Supplement: Supplementary file 2 [file Data_Sheet_2.PDF]

UPPSALA UNIVERSITET

Frågeformulär till vårdnadshavare:
Studie om barns språkutveckling

BiLI TAS [Bilingualism &Language Impairment | Turkish Arabic Swedish]

1. Barnets namn ___________________________________

2. Födelsedatum ___________________________________

3. Kön ☐ Flicka ☐ Pojke

4. Är det ditt första barn? Andra? Tredje? Kryssa rätt nummer

☐ 1 ☐ 2 ☐ 3 ☐ annat nummer ____

5. Vilka språk talar ditt barn nu?

☐ Svenska

☐ Arabiska, vilken dialekt?
☐ Irakisk ☐ Libanesisk
☐ Palestinsk ☐ Syrisk
☐ Annan: ______________

☐ Andra språk, vilka?
____

6. I vilket land föddes ditt barn?

☐ I Sverige

☐ I ett land där arabiska talas
Vilket?________________

☐ I ett annat land
Vilket?_______________

7. Hur länge har ditt barn bott i Sverige?

☐ Sedan födseln ☐ Annan tid: Antal år____

8. a) Går ditt barn i skolan?

☐ Ja ☐ Nej

Om ja:

Namn på skola:

____

Ditt barns ålder vid skolstart:___ År ___Månader

Vilken typ av skola?

☐ Enspråkig Svenska
☐ Tvåspråkig Svenska/Arabiska
☐ Annat ___________________________

Har ditt barn gått i förskola?

☐ Ja ☐ Nej

Typ av förskola

☐ Enspråkig Svenska
☐ Tvåspråkig Svenska/Arabiska
☐ Annat _______________

Ditt barns ålder vid förskolestart:

___År ___Månader

Vistelsetid per vecka: ______timmar

Har det varit längre uppehåll (t.ex. p.g.a. syskons födelse)?

☐ Nej ☐ Ja, _____ månader

Vilka språk talade personalen med ditt barn?

____

Om nej:

Går ditt barn i förskola?

☐ Ja ☐ Nej

Typ av förskola:

☐ Enspråkig Svenska
☐ Tvåspråkig Svenska/Arabiska
☐ Annat __________________

Ditt barns ålder vid förskolestart:

____År ____Månader

Vistelsetid per vecka: ______timmar

Har det varit längre uppehåll (t.ex. p.g.a. syskons födelse)?

☐ Nej ☐ Ja, _____ månader

Vilka språk talar personalen med ditt barn?

____

Namn på förskola:

____

8. b) Går ditt barn på fritids?

☐ Nej ☐ Ja

9. Hur upplever du att ditt barns språkutveckling har varit?

Arabiska ☐ Tidig ☐ Normal ☐ Sen

Svenska ☐ Tidig ☐ Normal ☐ Sen

10. Hur gammalt var ditt barn när hon/han sa sina första ord?

På arabiska
___år___månader

På svenska
___år___månader

11. Hur gammal var ditt barn när hon/han sa fler än ett ord i följd?

På arabiska
___år___månader

På svenska
___år___månader

12. Har du någon gång varit orolig för ditt barns språkutveckling?

☐ Nej

☐ Ja, när och varför?
____

13. Har någon i familjen haft språksvårigheter och/eller läs- och skrivsvårigheter?

☐ Nej

☐ Ja, på vilket sätt?
____

14. Tycker du att ditt barns språkutveckling skiljer sig från syskon, kusiner eller lekkamraters?

☐ Nej

☐ Ja, på vilket sätt?
____

15. Har ditt barn någon gång haft kontakt med logoped?

☐ Nej

☐ Ja
Om ja, varför? ___________________________

16. Har ditt barn haft hörselproblem?

Hörselnedsättning
☐ Nej
☐ Ja

Upprepade öroninflammationer
☐ Nej
☐ Ja, hur många? _________
☐ Mitt barn har rör

17. Hör ditt barn normalt nu?

☐ Nej ☐ Ja

18. Information om föräldrar/vårdnadshavare

**Språkkunskaper**	Är **arabiska** ditt modersmål? **Om nej**, hur väl talar du språket?	Är **svenska** ditt modersmål? **Om nej**, hur väl talar du språket?	Vilka andra språk talar du?
Vårdnadshavare Förälder 1			
Vårdnadshavare Förälder 2			

**Bakgrund**	Var föddes du? (Land, region)	Var växte du upp? (Land, region)	Hur länge har du bott i Sverige?
Vårdnadshavare Förälder 1			
Vårdnadshavare Förälder 2			

**Utbildning och yrke**	Utbildning	I vilket land har du genomfört huvuddelen av din utbildning?	Yrke
Vårdnadshavare Förälder 1			
Vårdnadshavare Förälder 2			

**19. Vilket språk talar <u>du</u> med ditt barn? *(sätt kryss)***	nästan bara arabiska	huvudsakligen arabiska, ibland svenska	arabiska 50%, svenska 50%	huvudsakligen svenska, ibland arabiska	nästan bara svenska	något annat:
Vårdnadshavare/ Förälder 1						
Vårdnadshavare Förälder 2						

**20. Vilket språk talar ni föräldrar/vårdnadshavare med varandra?**

**21. Vilket språk talar <u>ditt barn</u> med dig? *(sätt kryss)***	nästan bara arabiska	huvudsakligen arabiska, ibland svenska	arabiska 50%, svenska 50%	huvudsakligen svenska, ibland arabiska	nästan bara svenska	något annat:
Vårdnadshavare Förälder 1						
Vårdnadshavare Förälder 2						

22. Vilket språk talar ditt barn med sina syskon?

☐ Mitt barn har inga syskon ☐ Både svenska och arabiska ☐ Annat språk
☐ Mest arabiska ☐ Mest svenska _______________

23. Från vilken ålder började ditt barn höra svenska regelbundet? (kryssa på skalan)

födseln	1 år	2 år	3 år	4 år	5 år	6 år	7 år

24. Från vilken ålder började ditt barn höra arabiska regelbundet? (kryssa på skalan)

födseln	1 år	2 år	3 år	4 år	5 år	6 år	7 år

25. Hör ditt barn arabiska från…?

☐ Syskon
☐ Andra släktningar / vänner till familjen
☐ Sina kompisar
☐ Böcker
☐ TV / film / dator / musik / surfplatta /smartphone
☐ Annat __________________________

Hör ditt barn andra arabiska dialekter än den ni pratar hemma?

☐ja ☐ nej

Om ja: vilka?____

Var:____

Hör ditt barn modern standardarabiska (t ex barnprogram på tv?)

☐ ja ☐ nej

Om ja: var?____

26. Hur mycket hör ditt barn båda språken i sin vardag? Markera på skalan

Svenska 5%<br>Arabiska 95%	Svenska 20%<br>Arabiska 80%	Svenska 40%<br>Arabiska 60%	Svenska 50%<br>Arabiska 50%	Svenska 60%<br>Arabiska 40%	Svenska 80%<br>Arabiska 20%	Svenska 95%<br>Arabiska 5%

☐ Annat: ____

27. Vilket språk talar ditt barn bäst just nu?

☐ Arabiska ☐Svenska
☐ Båda lika bra ☐ Annat språk __________________

28. Tycker ditt barn mer om att tala ett av språken?

☐ Nej ☐ Ja, vilket? _______________________

29. Bedöm ditt barns språkkunskaper genom att markera ett alternativ

	Väldigt bra	Bra	Ganska bra	Dåligt	Mycket dåligt
Hur väl förstår ditt barn arabiska?					
Hur väl förstår ditt barn svenska?					
Hur väl talar ditt barn arabiska?					
Hur väl talar ditt barn svenska?					

30. Vilket är viktigast för dig att ditt barn lär sig …?

☐ Svenska är viktigast ☐ Båda lika viktiga
☐ Arabiska är viktigast ☐ Annat:_______________________________

31. Tar ditt barn del av någon typ av modersmålsundervisning/stöd i arabiska?

☐ Ja, kommunens modersmålslärare ☐ Nej
☐ Ja, i privat regi (t ex via föreningar eller religiösa samfund)

Om **ja**, ange antal timmar per vecka ______
Om **ja**, är det tillsammans med andra barn ☐ Ja ☐ Nej

32. Ägnar sig ditt barn åt organiserade fritidsaktiviteter (t.ex. sport, musik)?

☐ Ja ☐ Nej

Om **ja**, på vilket språk äger aktiviteterna rum?

	**Varje dag**	**Minst 1 gång i veckan**	**Sällan**
Arabiska			
Svenska			
Annat språk, ange vilket: _______________			

**33. Hur ofta har ni utfört följande aktiviteter med ert barn <u>under den senaste månaden</u>? *(sätt kryss)***	**Arabiska**				**Svenska**			
	Aldrig	Två gånger i månaden	En eller två gånger i veckan	Nästan varje dag	Aldrig	Två gånger i månaden	En eller två gånger i veckan	Nästan varje dag
Berätta historier								
Läsa böcker								
Lyssna på sånger eller sjunga								
Titta på TV / film / datorspel								

34. Brukar ditt barn vilja berätta sagor och historier för dig?

☐ Ja, ofta ☐ Ja, ibland ☐ Ja, men sällan ☐ Nej

35. Kan ditt barn läsa och skriva?

☐ Nej
☐ Ja, skriva och läsa sitt namn
☐ Ja, enklare ord/meningar på arabiska
☐ Ja, flytande på arabiska
☐ Ja, enklare ord/meningar på svenska
☐ Ja, flytande på svenska

36. Är det något du vill tillägga?

____

____

____

Tack för din medverkan!
